# Lactate Transporters in the Context of Prostate Cancer Metabolism: What Do We Know?

**DOI:** 10.3390/ijms151018333

**Published:** 2014-10-13

**Authors:** Nelma Pértega-Gomes, Fátima Baltazar

**Affiliations:** 1 Life and Health Sciences Research Institute (ICVS), School of Health Sciences, University of Minho, Braga 4710-057, Portugal; E-Mail: nelma.gomes@cruk.cam.ac.uk; 2 ICVS/3B’s—PT Government Associate Laboratory, Braga 4710-057, Portugal

**Keywords:** prostate cancer, monocarboxylate transporters, cancer metabolism, therapeutic targets

## Abstract

Metabolic changes during malignant transformation have been noted for many years in tumours. Otto Warburg first reported that cancer cells preferentially rely on glycolysis for energy production, even in the presence of oxygen, leading to the production of high levels of lactate. The crucial role of lactate efflux and exchange within the tumour microenvironment drew attention to monocarboxylate transporters (MCTs). MCTs have been recognized as promising targets in cancer therapy, and their expression was described in a large variety of tumours; however, studies showing how these isoforms contribute to the acquisition of the malignant phenotype are scarce and still unclear regarding prostate cancer. In this review, we focus on the role for MCTs in cell metabolism, supporting the development and progression of prostate cancer, and discuss the exploitation of the metabolic nature of prostate cancer for therapeutic and diagnostic purposes.

## 1. Introduction

Prostate cancer (PCa) is the most diagnosed malignancy in men and the second leading cause of cancer-related death in the USA [1]. PCa incidence rates over the past 30 years reflect changes in PCa detection. The PSA test increased dramatically in the U.S. in the late 1980s, with a peak of incidence in 1992. Recently, a decline in incidence rates among men of all ages was observed [2]. Despite PCa’s high morbidity, its etiology remains obscure, with the only established risk factors being increasing age, race and family history. Many putative risk factors, including hormones, dietary factors, obesity, physical inactivity, occupation, vasectomy, smoking, sexual factors and genetic susceptibility, have been implicated, but the epidemiologic evidence is not conclusive. While it is not known whether the risk factors explaining the observed patterns are environmental, lifestyle or genetic, it is likely that a complex interplay of these factors is associated with PCa development. Early detection in PCa is crucial, since only organ-confined disease is amenable to curative treatment, whereas patients with advanced disease can only be palliated. Although there are some available methods frequently used for PCa detection, their performance is sub-optimal due to non-satisfactory sensitivity and specificity rates. In this context, new therapeutic strategies, as well as new biomarkers are urgently needed, not only for early detection, but also as ancillary tools for diagnosis, which is still based on the histopathological evaluation of biopsy specimens [3,4,5,6,7].

## 2. Reprogramming of Energy Metabolism as an Emerging Hallmark of Cancer

Advances in cancer research have generated rich, but also extremely complex, knowledge, revealing cancer as a disease that involves several dynamic changes. In order to organize the complexity of cancer, the biological capabilities acquired during the multistep development of human tumours and shared by all cancers were grouped and denominated as the “hallmarks of cancer” to create an organized principle that provides a logical framework to understand the diversity of neoplastic disease [8]. Lately, new hallmarks have emerged and reprogramming of energy metabolism was considered an emerging hallmark of cancer, since it was recognized that chronic and uncontrolled cell proliferation, which represents the essence of neoplastic disease, involves also adjustments of energy metabolism to fuel cell growth and division [9].

Altered energy metabolism is being proven to be as widespread in cancer cells as many of the other cancer-associated traits that have been accepted as hallmarks of cancer. However, just recently, major relevance has been given to cancer metabolism. The observation that tumour cells exhibit an altered metabolism when compared to normal cells was made almost one century ago by the Nobel Prize winner, Otto Warburg, who described it as the first tumour specific-alteration. Warburg first observed that cancer cells can reprogram their glucose metabolism, and, thus, their energy production, by limiting their energy metabolism largely to glycolysis, leading to a state that has been called “aerobic glycolysis” [10]. Glycolysis, the first major pathway of cellular metabolism, occurs in the cytoplasm and is functional even in the absence of oxygen. While glycolysis is able to produce ATP at high rates, it is considered a low efficiency pathway, because it produces only two ATP molecules per glucose. In order to compensate for the lower efficiency in ATP production obtained by glycolysis relative to mitochondrial oxidative phosphorylation, cancer cells markedly increased uptake and utilization of glucose in many human tumour types, the rate of entry being higher than in normal cells [11,12,13].

Importantly, recent evidence showed that some tumours contain distinct subpopulations of cancer cells that differ in their energy-generating pathways. One subpopulation consists of glucose-dependent cells that consume high levels of glucose and secrete high levels of lactate, whereas the other subpopulation preferentially utilizes the lactate produced by the other cells as their main energy source, employing part of the citric acid cycle to do so. Thus, the hypoxic cancer cells rely on glucose for fuel and secrete lactate as waste; this lactate is then imported and preferentially used as fuel by better-oxygenated cells, creating an energetic symbiosis in which lactate is the major player, with advantages for the maintenance and progression of the tumours. The increased uptake of glucose by malignant cells has proven useful to detect tumours and also monitor their treatment, as it is the basis for the clinical use of positron emission tomography (PET) that uses a radiolabeled analogue of glucose (18F-fluorodeoxyglucose, FDG) as a reporter [14]. FDG is recognized as a substrate for glucose transport systems; thus, the rate of entry of this glucose derivate into cells is determined by the activity of glucose transport systems. Inside the cells, the accumulation of the radioactive glucose analogue serves as a read-out for the rate of glucose entry into cells. Since tumour cells exhibit enhanced glucose uptake compared to adjacent normal cells, PET is able to detect tumours and differentiate them from normal tissue [15,16]. Although the majority of metabolic cancer research focuses on the role of glycolysis, it has also recently become apparent that the tricarboxylic acid (TCA) cycle and oxidative phosphorylation (OXPHOS) also have major roles in many types of cancer, including PCa [11,17,18,19]. Studies suggest that increased citrate oxidation is a significant metabolic characteristic for the bioenergy requirement in PCa. The normal human prostate gland has the function of producing, accumulating and secreting high levels of citrate [20,21], and in contrast to normal glands and benign hyperplasia, PCa is characterized by low levels of citrate. Based on the bioenergetics of prostate epithelial cell metabolism, Costello and Franklin proposed the “bioenergetic theory of prostate malignancy”: The transformation of a citrate-producing sane epithelial cell to a malignant citrate-oxidising cell that would result in a more efficient energy-generating system. Additionally, the authors suggest that in order to meet the energetic requirements of malignant cells, the metabolic transformation into citrate oxidation must be an early event in preparation for the progression of malignancy and preceded the histopathologic identification of malignant cells [20,22,23]. In fact, changes of citrate levels in the form of choline/citrate for detection and localization of PCa is a basis of MRS (magnetic resonance spectroscopy for *in situ* detection of PCa [24,25]. Other studies showed that the activity of mitochondrial (m)-aconitase, the first reaction before citrate oxidation, is significantly higher in PCa compared to normal prostate, which drives the utilization of citrate as an energy source [20]. In order to maintain a sustained citrate production, the continuous availability of oxaloacetate and acetyl-CoA is required for continuous citrate synthesis. Acetyl-coenzyme A is the only molecule consumed in the citrate cycle, and its continuous availability is crucial for driving citrate oxidation. Studies also suggested that to meet the bioenergetic requirement for rapid cell proliferation in PCa, there are changes of fatty acid metabolism that provide both ATP and acetyl-CoA to make the acceleration of citrate oxidation possible [26,27]. Furthermore, the literature regarding the utility of PET scans for prostate cancer detection is controversial, indicating that PCa can exhibit unique metabolic profiles; however, the clinical impact of these metabolic profiles is not known.

The lactate that is produced as a consequence of increased glycolysis is largely associated with poor prognosis, disease-free survival and overall survival in several cancers [28,29,30]. The crucial role of lactate efflux and exchange within the tumour microenvironment drew attention to monocarboxylate transporters (MCTs), which transport monocarboxylates, such as lactate across the membranes, therefore, playing a central role in cellular metabolism and metabolic communication between tissues. Here, we discuss what is known so far about PCa metabolism, MCT expression in PCa tissues and the possibility to explore these lactate transporters for the development of novel diagnostic, prognostic and therapeutic strategies in the context of PCa.

## 3. Role of Monocarboxylate Transporters (MCTs) in Cellular Metabolism

The transport of monocarboxylates across the plasma membrane was originally thought to be via non-ionic diffusion of the free acid; however, the demonstration that lactate and pyruvate transport into human erythrocytes could be strongly inhibited after treatment with chemicals allowed the identification of a specific monocarboxylate transport mechanism. The transport of monocarboxylates was then characterized extensively in different cell types, and the observed characteristics led to the rationale for the existence of a family of monocarboxylate transporters [31,32]. MCTs are encoded by the SLC16 gene family, which is conserved among species, including rat, mouse, chicken and others. The family is presently composed by 14 members, which were identified through screening of genomic and expressed sequence tag databases. These proteins catalyse the transport of important monocarboxylates, namely pyruvate and lactate, with a proton, with no direct energy input involved in this process [31]. To function, an MCT translocates a proton and a monocarboxylate through the plasma membrane by an ordered mechanism in which H^+^ binding is followed by monocarboxylate binding to the protonated transporter [33]. Therefore, MCT activity is dependent on both, besides substrate concentration and the proton gradient between the extracellular and intracellular milieus. Lactate is indeed the monocarboxylate whose transport across the plasma membrane is quantitatively more important; however, MCTs are also important for the transport of many other metabolically important monocarboxylates, such as pyruvate, the branched-chain oxoacids derived from leucine, valine and isoleucine, and the ketone bodies, acetoacetate, β-hydroxybutyrate and acetate [34]. Besides being a family of 14 members, only the first four (MCT1–MCT4) have been demonstrated experimentally to facilitate the proton-linked transport of metabolically important monocarboxylates [33,35,36,37]. Since MCT3 is a very specialized MCT, being limited to the retinal pigment and choroid plexus epithelia [38,39], this review will only focus on MCT1, MCT2 and MCT4 isoforms, whose function is responsible for the name of this family of transporters. MCT1 has a broader distribution and transports a wider range of substrates when compared to other family members. The main function of this transporter has been associated with the uptake or efflux of monocarboxylates through the plasma membrane, according to cell metabolic needs and behaving as a high affinity transporter for l-lactate, but not for d-lactate, as well as for pyruvate, acetate, propionate, d,l-β-hydroxybutyrate and acetoacetate [36]. MCT2 displays a higher affinity for l-lactate, pyruvate, d-β-hydroxybutyrate and acetoacetate than MCT1. When expressed in the same tissue, MCT1 and MCT2 are located in distinct cells, as they have been suggested to play different roles in metabolic shuttles [40,41]. The physiological role of the human MCT4 is mostly associated with the export of lactate in cells with high glycolytic rates related to hypoxic energy production [42]. It was characterized by heterologous expression in *Xenopus laevis* oocytes, exhibiting the highest *K*_m_ values for most substrates and inhibitors when compared to MCT1 and MCT2 [43]. Although the regulatory mechanisms of MCT expression are not completely elucidated, evidence indicates that MCTs are regulated at both transcriptional and post-transcriptional levels. Importantly, hypoxia is known to be a major regulator of MCT expression. While there is some controversy around MCT1, evidence for MCT4 up-regulation by hypoxia is more consistent [44,45,46,47,48]. Actually, MCT4 was described to be regulated by the hypoxia inducible factor 1α (HIF-1α), a transcription factor with a major role in the adaptation to hypoxia [45]. MCTs are also regulated by interactions with ancillary proteins in order to be properly expressed in the cellular membrane. MCT1 and MCT4 are regulated by association with the cell surface glycoprotein CD147 (also known as basigin or EMMPRIN), while gp70 (EMBIGIN) is described for translocation of MCT2 to the plasma membrane [49]. CD147 does not exclusively act as a chaperone; in fact, it is a broadly distributed plasma membrane glycoprotein, which belongs to the immunoglobulin superfamily. CD147 is ubiquitously expressed on the cell surface, with the highest levels found in metabolically active cells [50,51,52]. Due to their crucial role in lactate efflux, MCTs have been recognized as promising targets in cancer therapy and described in a large variety of tumours.

## 4. Role of MCTs in the Context of Cancer

Regarding cancer research, there is already several pieces of evidence for the upregulation of MCTs in several solid tumours, such as colorectal carcinomas [53], uterine cervix carcinomas [54], glioblastomas [55], breast carcinomas [56], lung tumours [57] and ovarian cancer [58], pointing to an important role for these transporters in the maintenance of these malignancies. Importantly, MCT expression was associated with the expression of the multidrug resistance markers, multidrug resistance protein 1 (MDR1) and multidrug resistance-associated protein 2 (MRP2) [58]. Overall, the data available in the literature support the hypothesis of a major role of MCTs in the maintenance of the hyper-glycolytic and acid-resistant phenotypes, as adaptations to the hypoxic microenvironment. The up-regulation of MCTs in the plasma membrane of different type of tumours is an adaptive mechanism to allow continuous high glycolytic rates, by exporting the accumulating end-product, lactate, as well as to counteract cancer cell acid-induced apoptosis or necrosis. However, it is clear that this might not be the case for all tumour types, and in many cases, there are no functional studies showing the dependence of the tumours on MCT expression and activity. Thus, additional studies on MCT expression in other tumour types, confirmation of the results already published, as well as additional functional studies are needed to deeply understand the role of MCTs in cancer cell maintenance and aggressiveness and exactly in which cases these transporters could be used for therapy. Regarding PCa, which is our main focus in this review, few studies exist exploring the value of MCT expression for the PCa cells.

### Role of MCTs as Therapeutic Targets in Cancer

From the above description, taking into account that there is upregulation of MCTs in several tumours, inhibition of these molecules will certainly disturb cancer cell homeostasis, by interfering with monocarboxylate transport and pH regulation, and can be a useful strategy to explore in cancer treatment. There are several known classical MCT inhibitors, including: (1) aromatic monocarboxylates, like α-cyano-4-hydroxycinnamate (CHC); (2) bioflavonoids, like quercetin and phloretin; and (3) stilbene-derived compounds, such as 4,4-*O*-diisothiocyanostilbene-2,2-disulphonate (DIDS) and 4,4'-dibenzamidostilbene-2,2'-disulfonate (DBDS) [34]. The sensitivities to the inhibitors vary among the MCT isoforms, and this difference may result from the different accessory proteins required for MCT activity [49]. For example, MCT2 is more sensitive to CHC, DIDS and DBDS than MCT1, and MCT4 exhibits lower sensitivity than MCT1 for a range of inhibitors [43]. However, these inhibitors may target other molecules besides MCTs, and the study of the functional role of MCTs in cancer should benefit from the use of specific inhibitors. AstraZeneca recently developed a series of small molecule compounds, such as AR-C155858, which were demonstrated to be MCT1/2 isoform specific and are presently in clinical trials [59].

The effect of MCT inhibition in cancer has been demonstrated using several models of cancer, including colorectal, cervix [47], gliomas [60], melanomas [61] and breast cancer [62]. Importantly, the classical inhibitor CHC was able to enhance the effect of temozolomide, a gold standard drug currently used in the treatment of gliomas [55]. Inhibition of MCT1/2 with the AstraZeneca inhibitor AR-C155858 inhibited lactate export, glycolysis rates and tumour growth using RAS-transformed fibroblasts. However, cells became resistant to MCT1/2 inhibition, and tumourigenicity was restored when MCT4 was expressed [63]. To more accurately study the role of MCTs in cancer, and taking into account that most inhibitors are not specific, the authors used several approaches to down-regulate MCTs, including RNA interference (RNAi) technology. MCT silencing decreases lactate flux and migration of glioma [55,64] and breast cancer cells [65,66] and decreases glycolytic flux and reduced tumour growth using human colon adenocarcinoma cells [63]. More recently, Kim *et al.* showed that MCT1 inhibition by CHC did not reduce tumour volume in prostate cancer, although there was an increase in necrotic tissue [67].

## 5. Diagnostic and Prognostic Value of MCTs in Prostate Cancer

### 5.1. Clinico-Pathological Significance of MCTs and CD147 Expressions in Prostate Carcinoma

As stated before, MCTs are transmembrane proteins involved in the transport of important monocarboxylates. To ensure the rapid efflux of lactate, most cancer cells express high levels of MCTs. It has long been recognized that MCTs might represent good targets for chemotherapy, and several *in vitro* studies have shown the potential of this approach; however, MCT regulates PCa during PCa progression, and the result of targeting MCTs in PCa is still largely unknown. Recently, Hao and co-workers [68] showed that overexpression of CD147, CD44v3-10, MDR1 and MCT4 was associated with PCa progression and also that expression of both CD147 and CD44v3-10 correlates with drug resistance during PCa metastisation and could be a useful potential therapeutic target in advanced disease. The authors showed co-localisation of CD147 and CD44v3-10 with MDR1 and MCTs in tumour and stromal cells, suggesting a role for these invasive markers in the regulation of drug resistance in the progression of PCa. This indicates that both CD147 and CD44v3-10 may be potential therapeutic targets for treating late-stage, incurable, recurrent metastatic PCa to overcome drug resistance.

**Figure 1:**
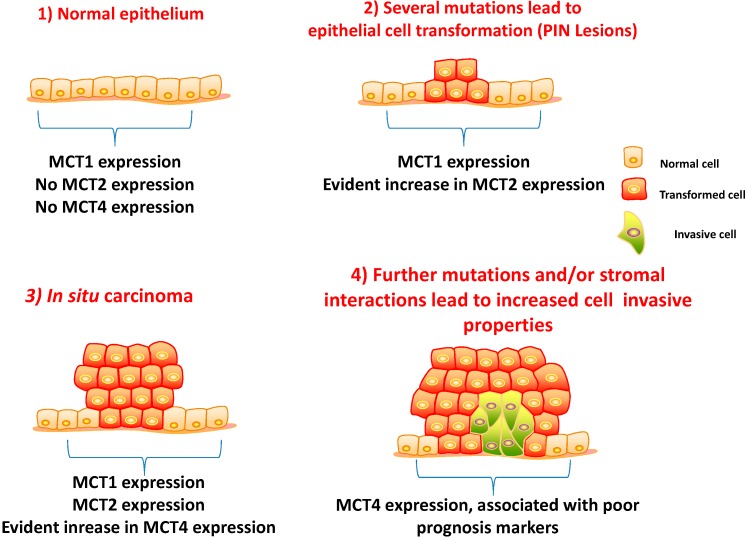
Schematic representation of the different stages of prostate cancer (PCa) tumour initiation and progression. Monocarboxylate transporter 1 (MCT1) is expressed in both non-neoplastic and malignant glands, whereas MCT2 is increased from benign to prostate intraepithelial neoplasia (PIN) lesions and malignant glands. MCT4 is only expressed in malignant glands, and its expression is associated with poor prognosis for patients.

Another previous study focusing on MCTs and CD147 expression in a well-characterized series revealed that at variance with other solid tumours, MCT1, MCT4 or CD147 (MCT1/MCT4 chaperone) were not found to be up-regulated at the plasma membrane of PCa cells [69]. In this work, some contribution was also made to understand MCT regulation by chaperones. Firstly, the regulation of MCT1 and MCT4, but not MCT2, by CD147 was supported by evidence from human tissues. Importantly, there was undetected expression of gp70 in PCa samples, suggesting that a as of yet identified chaperone could be involved in MCT2 trafficking. However, there was an increase in both MCT2 and MCT4 expressions observed from non-neoplastic (normal or adjacent) to tumour tissues, accompanied by a decrease in MCT1 and CD147 expressions in the transition from normal or adjacent non-neoplastic tissue to PCa (Figure 1). These observations suggest that MCT1 may have an important role in normal tissue, where it is highly expressed and is downregulated in PCa cells, where other adaptive mechanisms may be activated. In contrast, the upregulation of MCT2 and MCT4 in the cytoplasm of cancer cells, with a granular appearance, suggested the presence of MCTs in organelle membranes. These findings are interesting and may suggest either the existence of alternative mechanisms that ensure acid efflux and the maintenance of intracellular pH or the presence of alternative metabolic pathways different from glycolysis that predominate in PCa.

Importantly, associations between MCT4 and CD147 expressions and reliable poor prognosis markers of disease progression, namely the Gleason score, pT3 stage of the tumour and biochemical recurrence after surgery, were observed [69]. This finding is of great importance, as the tumours that present these characteristics have a more aggressive clinical behaviour and, until now, do not have a specific molecular therapy. Additionally, in the same study, MCT2 was found to be highly expressed in the cytoplasm of PCa specimens and prostate intraepithelial neoplasia (PIN) lesions, however, with no clinico-pathological associations, suggesting a role in malignant transformation more than in the disease aggressiveness.

Although important correlations were found, the presence of MCT1 in both tumour cells and non-malignant tissues, as well as the absence of MCT4 at the plasma membrane led to the hypothesis that PCa might rely less than the majority of tumours on aerobic glycolysis.

### 5.2. MCT2 as a Putative Prostate Cancer Biomarker

**Figure 2:**
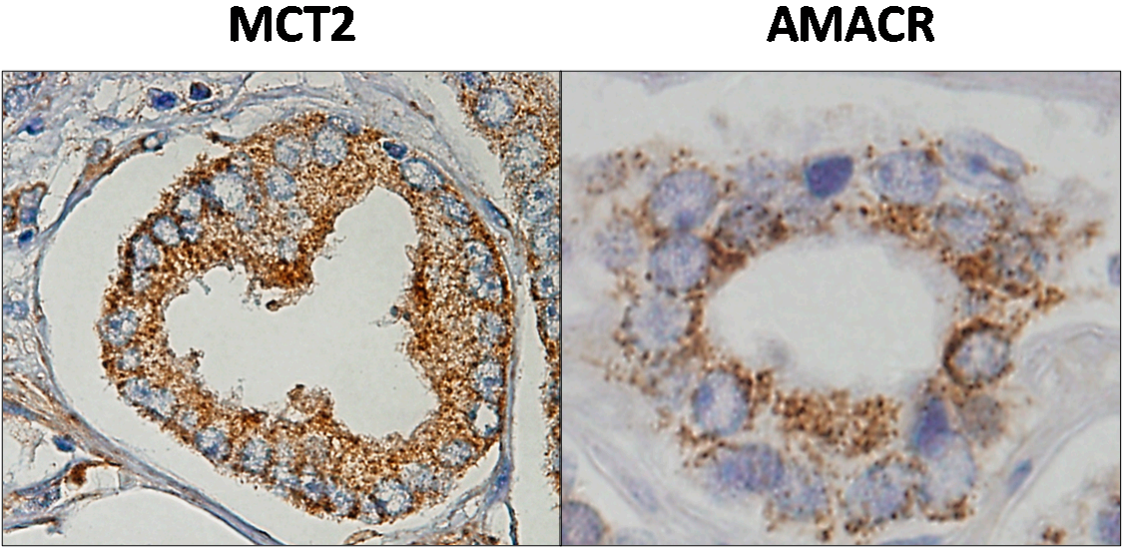
MCT2 and α-methylacyl-CoA-racemase (AMACR) expression in a prostate malignant gland by immunohistochemistry. Both proteins exhibited a punctuated staining in the cytoplasm of malignant cells.

The sensitivity and specificity exhibited by MCT2 to recognize PCa was further analysed and compared with the immunohistochemical expression of α-methylacyl-CoA-racemase (AMACR) in a large series of prostate samples and also measuring the sensitivity and specificity of combining both as positive markers with the negative markers p63 and 34βE12 [70]. In fact, immunohistochemistry revealed that, like AMACR, MCT2 overexpression occurs in virtually all Gleason grades with a predominance of diffuse overexpression, with more than 50% of tumour stained in positive cases, meaning that the positivity of MCT2 is also independent of the Gleason score. Figure 2 shows the expression of MCT2 and AMACR in a PCa malignant gland.

Analysing the results of the triple combination, we observed that the use of two positive markers (AMACR/MCT2) with one negative marker (p63 or 64βE12) instead of one positive marker with two negative markers improves the sensitivity to detect PCa, as well as the negative predictive value, decreasing the possibility of diagnosing benign prostate tissue as PCa. Furthermore, the observation that MCT2 also stains strongly in PIN lesions indicates that these two proteins may be involved in tumour initiation. However, as well as AMACR, MCT2 expression was not correlated with poor prognosis parameters, but it was present in high grade PIN, suggesting a possible role in the malignant transformation. It is important to note that MCT2 is one of the least explored MCT isoforms in cancer. This could be due to the fact that only a few tumours exhibit a high expression, and it has not been reported at the plasma membrane. Thus, MCT2 does not appear to be involved in the transmembrane transport of lactate in/out of the cell, but it may have other, no less important, functions that need to be unveiled. However, further studies are needed to clarify the role of both markers on PCa initiation/progression. Being that MCT2 is highly expressed in the localized tumour and MCT4 in only the most aggressive tumours, this was indicative that the carcinogenic process leading to metastatic tumour may require metabolic adaptations involving specific MCT upregulation and specific subcellular localization across different stages of the disease.

### 5.3. A Prognostic Value for a Lactate Shuttle Established between Prostate Cancer Cells and Cancer Associated Fibroblasts

**Figure 3:**
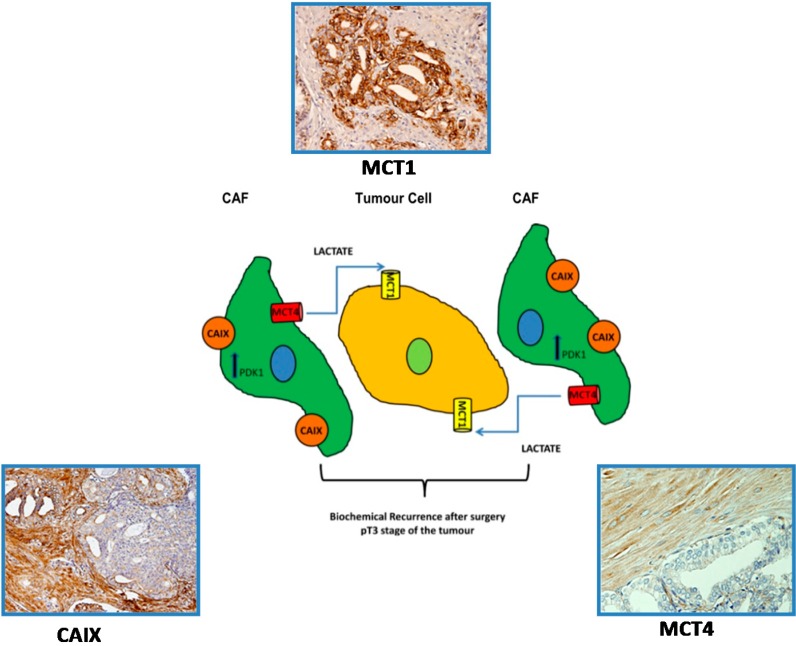
Schematic representation of the lactate shuttle system between malignant cells and cancer associated fibroblasts (CAFs). The expression of MCT4 in CAFs together with the expression of MCT1 in tumour cells is associated with biochemical recurrence after surgery and the pT3 stage of the tumour. Adapted from [70].

MCTs shall have a vital role in the emergence of both the hyper-glycolytic and acid-resistant phenotypes by enabling lactate efflux from cancer cells, as well as regulating the intracellular pH. In light of the microenvironmental model of carcinogenesis, it is proposed that lactate release from glycolytic/hypoxic fibroblasts occurs through the low-affinity lactate transporter MCT4, and lactate uptake by the oxidative/oxygenated cancer cells occurs through the high-affinity MCT1 transporter. In cancer models, studies by Lisanti M.P. and collaborators described the importance of cancer-associated fibroblasts in fuelling and sustaining tumour growth and the importance of MCTs in this context [71,72], namely in breast cancer [73] and B-cell lymphoma [74]. In prostate cancer, Fiaschi T. *et al.* described that prostate cancer cells gradually became independent of glucose consumption, accompanied by a dependence on lactate uptake, to drive anabolic pathways for cell growth. Supporting these findings, the authors showed that pharmacologic inhibition of MCT1-mediated lactate uptake substantially decreased prostate cancer cell survival and tumour growth. In conclusion, the authors state that cancer cells allocate Warburg metabolism to their “corrupted” cancer associated fibroblasts (CAFs), using their by-products to grow in low glucose levels, in a symbiotic model in which stromal cells adapt to glucose availability [75]. Recently, Sanita P. *et al.* showed that PCa progression may benefit from MCT1 expression in tumour cells and MCT4 in the tumour-associated stromal cells. Thus, MCTs may be promising therapeutic targets in different phases of neoplastic transformation following a strategy aimed to contrast the energy metabolic adaptation of PCa cells to stressful environments [76]. In our studies, we observed that prostate cancer cells did not rely mainly on glycolytic metabolism, while there was a high expression of MCT4 and carbonic anhydrase IX (CAIX) in CAFs. This corroborates the hypothesis of the “Reverse Warburg effect” in prostate cancer, in which fibroblasts are under oxidative stress and express CAIX, an established hypoxia marker. We found that alterations in the expression of metabolism-related proteins were already evident in the early stages of malignant transformation, suggesting the continuing alteration of CAFs from an early stage. Importantly, the clinico-pathological significance of this lactate shuttle appears to be linked to poor prognosis parameters, namely the presence of biochemical recurrence after surgery, suggesting that immunohistochemical detection of proteins involved in the lactate shuttle may potentially prove to be useful as prognostic markers (Figure 3) [77].

## 6. Targeting Metabolism in Prostate Cancer: Is There a Therapeutic Window?

As stated before, PCa energetic metabolism appears to be unique in comparison with other types of solid cancers. Normal prostate cells mainly rely on glucose oxidation to provide precursors for the synthesis and secretion of citrate, resulting in an incomplete Krebs cycle and minimal oxidative phosphorylation for energy production. In contrast, during transformation, PCa cells no longer secrete citrate, and they reactivate the Krebs cycle as the energy source. Moreover, primary PCas do not show increased aerobic glycolysis, and therefore, they are not efficiently detectable with an 2-deoxy-2[F-18]fluoro-d-glucose positron emission tomography (FDG-PET) scan. PCa is not as glycolytic as the majority of other cancers, and increased glycolysis is found mainly in the advanced stages of the disease. On the other hand, an increase in fatty acid synthesis seems to be an early event in PCa tumorigenesis and is correlated with the progression of the disease [78,79]. Consequently, it is still controversial which metabolic pathway represents the most appropriate target for metabolic inhibition in PCa [79]. Although some evidence supports a crucial role of fatty acid-related metabolism in the pathogenesis and progression of prostate malignancy [27,68], reports are scarce with a low number of clinical samples and with no information regarding the clinico-pathological significance of these alterations. In contrast to other tumours, there are no drugs targeting metabolic pathways in PCa. A recent study using *in vivo* models showed that there were no significant differences in tumour volumes after treatment with an MCT1 inhibitor [67]. In addition, the group of Fiaschi *et al.* showed that pharmacological inhibition of MCT1 activity significantly affected prostate carcinoma cell survival and tumour outgrowth [75]. Thus, the real value of targeting MCTs in Pca is still largely unknown.

## 7. Concluding Remarks

It is known that different MCT isoforms are differentially expressed across PCa progression and possibly accordingly to the demands of PCa cells at each stage. The observation that MCTs are not restricted to the plasma membrane of prostate cells indicates their involvement in alternative cellular roles different from glycolysis. Data obtained so far point to the presence of different metabolic phenotypes across malignant transformation, in which different isoforms of MCTs seem to be involved in different stages of PCa progression. On the one hand, MCT1 and MCT2 seem important in the maintenance of localized disease, whereas MCT4 is related with an aggressive phenotype, leading to the idea that different MCTs should be targeted across PCa disease progression. Much was achieved so far, but many other doors are now open that should be explored. Besides the more obvious lines that can be further explored, other directions can be taken, such as the study of other metabolic pathways, like glutaminolysis, microenvironmental conditions, like acidity and hypoxia, and other players in MCT regulation, such as HIF-1α, Akt c-myc and others. Importantly, since cell culture does not mimic all real tumour conditions, including O_2_ and nutrient limitation, key factors in metabolism, it is fundamental to assess the effects of MCT inhibition *in vivo*, evaluating aggressiveness parameters, such as tumour growth, angiogenesis and metastisation. In parallel, as MCTs are also important in physiological homeostasis, toxicity studies to determine MCT inhibition side effects will determine the actual potential of MCTs as therapeutic targets in cancer.

In conclusion, the observations discussed in this review demonstrate an important role for the metabolic demands of PCa tumours during disease progression in which MCTs play an important role and might represent promising therapeutic targets in different phases of neoplastic transformation and progression. This encourages the exploitation of MCTs as potential targets for PCa therapy and paves the way for further efforts to understand the role of MCTs in solid tumours, such as PCa, which does not appear to rely mainly on glycolytic metabolism for energy production. Although major advances have been made, many other studies are needed to complement the present knowledge on the role of MCTs in PCa survival and aggressiveness.
